# Influences of Western Cape community service doctors’ choice regarding public, rural practice

**DOI:** 10.4102/safp.v67i1.6125

**Published:** 2025-07-15

**Authors:** Tamryn J. Baytopp, Vanessa Lomas-Marais, Ts’epo Motsohi

**Affiliations:** 1 Department of Family Medicine and Primary Care, Faculty of Health Science, Stellenbosch University, Cape Town, South Africa

**Keywords:** community service, rural, primary health, district health, employment, public practice, Western Cape, physician

## Abstract

**Background:**

Staff shortages in rural areas have led to unequal healthcare access in South Africa. The compulsory community service programme aims to address this disparity; but to be effective, it must encourage doctors to remain in rural facilities beyond their service periods. Identifying factors that influence their decisions to stay is crucial for developing strategies to improve rural doctor retention. The aim is to describe the important factors influencing Western Cape community service doctors’ choice of whether they will seek employment in public rural practice.

**Methods:**

An observational cross-sectional study with correlational analysis was conducted using an internet-based questionnaire. This study was conducted on community service doctors who were employed in the Western Cape in 2022.

**Results:**

Eighty-six doctors participated, with 8% intending to work in rural practice in 2023 and 21% considering it in the future. Significant factors associated with rural practice intentions included rural upbringing (6.5 times more likely), rural internship placement (7.7 times more likely) and rural community service (3.5 times more likely). Key influences were personal safety, job satisfaction and mental health.

**Conclusion:**

The proportion of doctors considering rural practice remains low. Policy revisions should focus on preferentially enrolling medical students with rural backgrounds and placing community service doctors in rural areas, alongside efforts to create safe, satisfying work environments that support mental health.

**Contribution:**

This study enhances the understanding of retaining healthcare professionals in underserved rural areas, addressing primary healthcare challenges in the African context.

## Introduction

There has been a long-standing global problem of inequitable access to healthcare in rural areas compared to urban areas.^1^ A large contributor to this problem is the shortage of healthcare workers in rural areas.^1^ In 2014, 52% of the global rural population did not have access to healthcare because of inadequate staffing, compared to 24% in urban areas.^2^ Globally, the maternal mortality ratios are about three times lower in urban areas than in rural areas; there is a higher rate of incomplete immunisation, and breast cancer is diagnosed at a more advanced stage in rural areas than in urban areas.^2^ The African continent also suffers from this disparity in the distribution of healthcare workers, with 75% of the Tanzanian population being rural dwellers, served by 26% of their doctors.^3^

South Africa faces the same challenge of inequitable health access between urban and rural areas. Provinces with smaller rural populations have a significantly higher number of doctors per capita than provinces with predominantly rural populations.^4^ Although 43% of South Africa’s population lives in rural areas, only 12% of doctors work in these areas.^4,5^ The disparity in healthcare access and quality between rural and urban areas can be evidenced by indicators such as the infant mortality rate. In 2007, the South African urban infant mortality rate was 43.2 per 1000 live births, while in rural areas it was 71.2 per 1000 live births.^6^ Case fatality for children < 5 years-old with severe acute respiratory illness was higher in rural areas than in urban areas.^7^

In response to this challenge, the South African National Department of Health has implemented several strategies to improve access to healthcare by attempting to improve human resources. Some of these strategies include the Human Resources for Health Strategy for the Health Sector: 2012/2013–2016/2017 (which suggests making more senior posts preferentially available in rural areas and re-establishing the generalist doctor as part of the PHC team), compulsory community service, increasing the number of healthcare workers trained and providing provincial bursaries to trainees.^4,5^

In 1998, South Africa introduced a compulsory 12-month community service period for all newly qualified doctors in the year following the completion of their internship training. In subsequent years, the programme was expanded to include dentists, nurses and other healthcare workers. The programme aimed to improve health services and help young healthcare workers with their professional development.^8^ In a review of the first 15 years of this community service programme, it was found that there has been a significant increase in rural placements, and now the programme ensures that around 8000 new healthcare workers enter the public service in their community service year.^8^ However, poor retention of staff following their community service means that this programme is yet to address the needs of the rural areas sustainably.^8^

Given the global nature of this problem, there is a significant body of research exploring factors related to health staff retention in rural areas. In both higher-income and lower-income settings, several key factors emerge repeatedly in the research literature.^3,9,10,11,12,13,14,15,16,17,18,19^ These factors can be divided into broad categories, including demographic, financial, professional or career, working and living conditions, as well as personal and social factors.^9,20^ A systematic review has found that men were more likely to serve rural communities, and most studies showed older doctors were more likely to work in rural areas.^9^

There have been some recent South African publications on rural health staff retention. A small qualitative study of 10 rural doctors in Limpopo found that hospital infrastructure, accommodation, continued education, specialist support, career progression, remuneration, management and community relationships are all significant factors.^20^ In a quantitative descriptive study conducted in three rural Northern KwaZulu-Natal Hospitals, 68.5% of doctors had burnout. This burnout was a significant deterring factor from their continued practice at these hospitals.^21^ This is comparable to the rest of sub-Saharan Africa, where 81% of rural healthcare workers were found to be burned out.^19^ High rates of depression and anxiety were also found among doctors in this area, and this was associated with a higher likelihood of planning to leave employment in the public service completely.^21^ In other South African studies, staff satisfaction and financial considerations were also found to influence staff retention and quality of health service provision in both urban and rural facilities.^13,14^

The 15-year review of the compulsory community service year for South African healthcare workers found that while the programme had managed to ensure more young doctors are deployed to rural or underserved institutions, the number of doctors planning to remain in rural practice did not increase and remained at approximately 15%.^8^ About 50% hoped to specialise, by training in predominantly urban centres, while the small number hoping to emigrate declined over the 15 years.

Although studies have been conducted to explore factors affecting doctors’ decisions to work in rural practice, there is currently insufficient quantitative data to explain these factors. Furthermore, there are even fewer studies on factors that influence the career decisions of community service doctors specifically.^20^ The aim of this study was to describe quantitatively the important factors influencing Western Cape community service doctors’ choice of whether they will seek employment in public rural practice.^22^

## Research methods and design

### Study design

This was an observational cross-sectional study with a correlational analysis of community service doctors working in the Western Cape in 2022.

### Setting

This study took place in the Western Cape province of South Africa. In the 2001 census, 9.6% of the population was classified as rural.^23^ The province’s district health management is divided into the Metro and Rural Health Services, with the Metro Health Services covering the City of Cape Town District, and Rural Health Services encompassing the remaining five districts. There are urban centres within the five rural districts. Therefore, for this study, rural healthcare institutions are defined as those in which the employees receive a rural allowance, and not whether the institution is under the management of Rural Health Services. Institutions receiving rural allowance include those identified by the National Executive as ‘rural development nodes’ as well as those classified as ‘rural’ during the bargaining of government and public service unions in 2004.^24^

### Study population

There were 207 community service doctors working in the Western Cape in 2022, and they were invited to participate through the Department of Health Human Resource official responsible for community service doctors provincially.

### Sample size

Based on a prior study on community service doctors in South Africa, 15% of these doctors planned to work in a rural setting at the completion of their year.^8^ Based on these estimates, it was calculated that a sample size of 101 would be required to achieve a 95% confidence interval and a 5% margin of error. This would amount to a response rate of 48.8%.

### Sampling

An attempt was made to invite the entire study population to participate to minimise sampling bias. Multiple reminders were sent to minimise non-responses. To reduce response bias, an informed consent section was provided, which emphasised that participation was anonymous and would not disadvantage the participant in any way. A Likert scale based on level of importance instead of ‘agree or disagree’ was used to avoid acquiescence bias. Of the 207 email invitations sent, 5 were not delivered to the recipients. Of the 202 invited participants, 97 opened the questionnaire, amounting to a response rate of 47.5%. Consent to participate was not given by five participants, and one respondent was excluded because they were not a community service doctor in the Western Cape in 2022. Moreover, five participants did not complete the demographics section of the questionnaire and were also excluded. This left a total of 86 complete participant records.

### Data collection

A self-administered, internet-based questionnaire was developed using REDcap software. It included multiple-choice and binary questions for demographic information, Likert Scale questions assessing the importance of different factors and a final open-ended question to include factors not included in the questionnaire. These factors were derived from those highlighted in existing international literature as well as those elicited in the qualitative study done in Limpopo, along with further additions by the researcher.^3,8,9,10,11,12,13,14,15,16,17,20,21^

The questionnaire was validated by an expert panel of three family physicians with experience in research and rural practice, a senior family medicine registrar with a rural employment history, as well as a community service doctor in a rural hospital. A pilot study was performed on the community service doctors working at Helderberg Hospital in 2022 using the email distribution and reminder strategy as intended for the main study. The pilot participants were asked to complete the same questionnaire 2 weeks apart to assess similarity in responses. A feedback form was completed by participants to assess time, question clarity and how well they felt their responses represented their actual feelings and thought processes. Two of the questions were adjusted based on participant feedback.

Data collection commenced in December 2022 and was completed in March 2023. An email was sent to the community service doctors notifying them of the study and its potential value in improving the appeal of working in rural hospitals in the future. There was a link to the survey attached for those who wished to participate.

### Data analysis

The data were captured from REDcap onto the Statistical Package for Social Sciences version 29.0 used for analysis. Data were then checked and analysed by the primary investigator with the assistance of the research supervisor and a biostatistician. Descriptive statistics were used for categorical data which were reported as frequencies. Numerical data were reported using medians and interquartile ranges or means and standard deviations depending on their distribution. To determine the statistical significance of the association between a demographic factor and future career in rural areas, the Fisher’s exact test was used for 2 × 2 tables and the Chi-square test for the unbalanced tables. Forward stepwise binary logistic regression was then used with a 10% cut-off to assess the association between the statistically significant associated demographic factors (*P* < 0.05). The model used to achieve this was tested using the Omnibus test and the Hosmer and Lemeshow test and was shown to be good (*p* = 0.746). For the Likert scale data, the mean of the answers was determined and ranked. Further analysis was conducted by sorting the individual Likert questions into groups based on the broader topics in the questionnaire. The cumulative mean of the questions in each category was then calculated. A basic narrative description was made of the data acquired in the final open-ended question.

### Ethical considerations

An application for full ethical approval was made to the Stellenbosch University, Health Research Ethics Committee and ethics consent was received on 06 October 2022. The ethics approval number is S22/07/121. Permission to conduct the study was also granted by the Western Cape Department of Health. All participants gave informed consent as described in the methods. Each online questionnaire was anonymised with a unique identifier. Data were stored in a password-protected file on REDCap and on OneDrive.

## Results

### Demographics of the participants

Table 1 summarises the participants’ characteristics. The majority of the participants were female (80.2%) and English-speaking (60.5%). The median age of participants was 28 years (Interquartile Range [IQR]: 26 to 30).

**TABLE 1:** Demographic characteristics of participants (*N* = 86).

Variable	Category	*n*	%
Gender	Male	17	19.8
	Female	69	80.2
Age (years)	26–30	73	84.9
	31–35	11	12.8
	35–40	2	2.3
Home language	Afrikaans	30	34.9
	English	52	60.5
	isiXhosa	3	3.5
	Other	1	1.2
Marital status	Single	38	44.2
	Married	43	50.0
	Life partner	4	4.7
Dependants	Yes	14	16.3
	No	71	82.6
	Unknown	1	1.2
Dependant type	Children	10	11.6
	Parents	4	4.7
	Siblings	3	3.5
	Other relatives	2	2.3
	Other	2	2.3
Place of origin (country)	South Africa	85	98.5
	Foreign national	1	1.2
Place of origin (urban or rural)	Urban	77	89.5
	Rural	9	10.5
Rural exposure in undergraduate studies	-	75	87.2
Rural exposure in internship	-	10	11.6
Rural exposure in community service	-	27	31.4

### Choice of future employment

Only 8% of participants were planning to remain in public rural practice in 2023, while 21% of participants indicated that they would consider it in the future. Thirty-six per cent of participants were certain that they would not consider rural practice and the other 43% were unsure whether they would consider working in public rural practice (Table 2).

**TABLE 2:** Participants’ plans to work in public rural practice in the future (*N* = 86).

Variable	Category	*n*	%
Participants’ plans to work in public rural practice in 2023	Yes	7	8.1
	No	79	91.9
Participants’ plans to work in public rural practice in the future	Yes	18	20.9
	No	31	36.0
	Unsure	37	43.0

### Association between participants’ demographic characteristics and the intention to work in public rural practice

Being placed in a rural area for community service and a rural upbringing were the only demographic factors found to have a statistically significant association with planning to work in public rural practice in 2023. In addition, growing up in a rural area and being placed in a rural area for internship and community service were found to have a statistically significant association with planning to work in public rural practice sometime in the future.

Based on Table 3, the statistically significant factors were further analysed for association using binary logistic regression. This confirmed that there was statistical significance in the association between rural upbringing and exposure during community service to an intention to work in rural practice in 2023. These associations, as well as rural exposure in internship, confirmed intention to work in rural practice in the future (see Table 4).

**TABLE 3:** Significance of association of variables to future plans to work in rural practice (Bivariate analysis).

Variable or category	Proportion of category with plans to work in public rural practice in 2023		*p*-values	Proportion of category with plans to work in public rural practice sometime in the future		*p*-values
	*n*	%		*n*	%	
**Gender**						
Male	2	28.6	0.620†	5	27.8	0.340†
Female	5	71.4		13	72.2	
**Home language**						
Afrikaans	4	57.1	0.170‡	7	38.9	0.206‡
English	2	28.6		9	50.0	
isiXhosa	1	14.3		2	11.1	
Other	0	0.0		0	0.0	
**Marital status**						
Single	4	57.1	0.290‡	6	33.3	0.550‡
Married	2	28.6		11	61.1	
Life partner	1	14.3		1	5.6	
**Dependants**						
With dependants	2	28.6	0.320†	3	16.7	0.980†
Without dependants	5	71.4		15	83.3	
**Place of origin (country)**						
South African citizen	7	100.0	1.000†	18	100.0	1.000†
Foreign national	0	0.0		0	0.0	
**Origin (upbringing)**						
Rural	3	42.9	0.020†**	5	27.8	0.007†
Urban	4	57.1		13	72.2	
**Rural exposure in undergraduate studies**						
No exposure	0	0.0	0.590†	3	16.7	0.692†
Some exposure	7	100.0		15	83.3	
**Rural exposure in internship**						
No exposure	5	71.4	0.190†	12	66.7	0.005†
Some exposure	2	28.6		6	33.3	
**Rural exposure in community service**						
No exposure	1	14.3	0.003†**	7	38.9	0.004†
Some exposure	6	85.7		11	61.1	

† , Fisher Exact test used.

‡ , Chi-Square test used.

** , Significant values.

**TABLE 4:** Association between statistically significant factors for future work in rural areas using binary logistic regression.

Factor	Likelihood of choosing to work in public rural practice in 2023 compared to those with urban exposure (95th CI)			Likelihood of choosing to work in public rural practice in the future compared to those with urban exposure (95th CI)		
	Likelihood	95% confidence interval	*p*	Likelihood	95% confidence interval	*p*
Rural upbringing	6.6	0.986–44.261	0.05	6.5	1.3–32.7	0.02
Rural exposure in internship	N/A	N/A	N/A	7.7	1.6–36.5	0.01
Rural exposure in community service	14.1	1.500–129.500	0.02	3.6	1.1–12.0	0.04

CI, confidence interval; N/A, not applicable.

### Factors influencing participants’ decision to work in public rural practice

**FIGURE 1:**
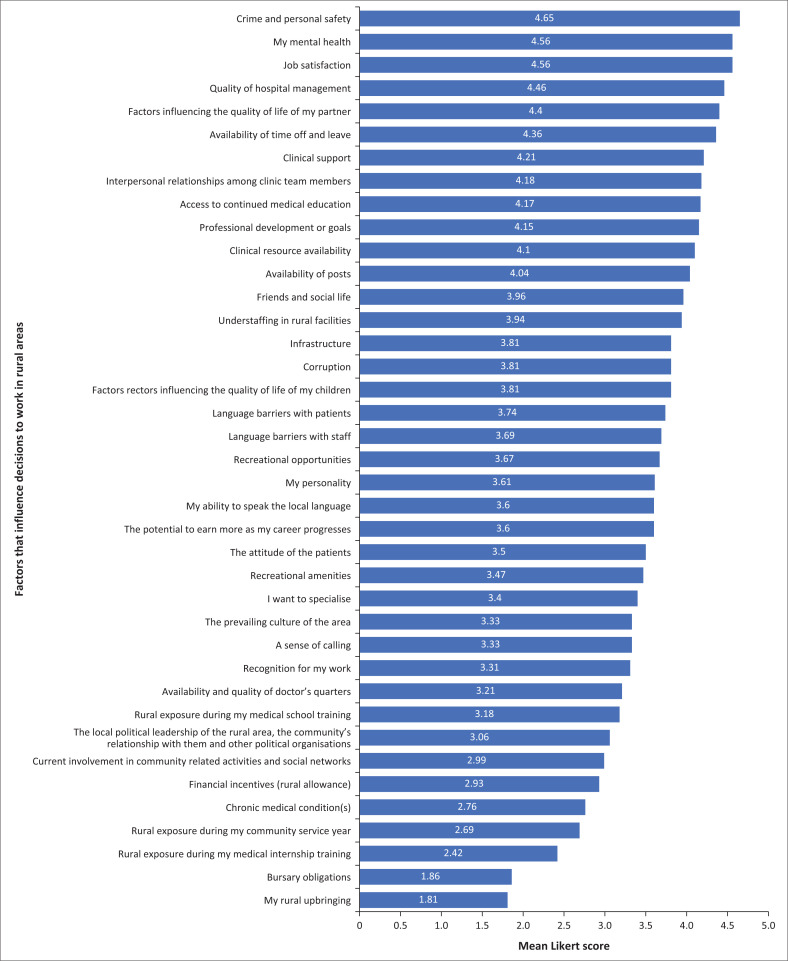
Importance of factors influencing decisions to work in rural areas.

Participants were asked to rank 39 factors based on their importance in deciding whether to work in public rural practice. The ranking used a Likert score of 1–5. Ten of the participants did not complete all of the Likert questions. For this reason, each question was analysed based on the total number of responses to it. The 39 factors that participants ranked by importance as influencing their decision are shown in Figure 1. The ranks are determined by their mean scores.

Participants rated ‘crime and personal safety’ as the most important factor in their decision whether to work in rural areas or not, with a mean Likert score of 4.65. This was closely followed by ‘job satisfaction’ and ‘my mental health’ both with mean scores of 4.56. The least influential or important factors were rural upbringing (1.81), bursary obligations (1.86), rural exposure in internship (2.42) and rural exposure in community service (2.69). Of the participants, 9 grew up in rural areas and their mean score for the influence of ‘rural upbringing’ was 3.125. The 10 participants who had rural exposure in internship had a mean score for the influence of ‘rural exposure during internship’ of 3.0. There were 27 participants who had rural exposure in community service and their mean score for the influence of ‘rural exposure during community service’ was 3.92. Therefore, those who had rural exposure did attribute more importance to these factors than those who did not.

**FIGURE 2:**
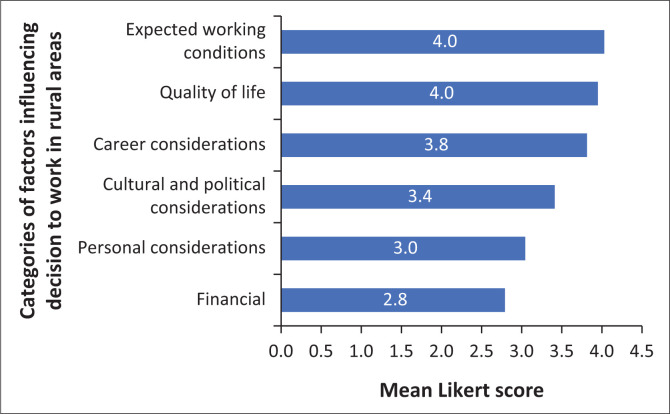
Importance of factors influencing decisions to work in rural areas by category.

The most influential category was ‘expected working conditions’ and the least influential was ‘financial considerations’ (see Figure 2).

### Free format question

Several factors which had not been covered in the Likert scale questions were identified. The two most frequently mentioned were proximity to family and proximity to an airport. Others included a preference for private practice, work-life balance, role models in rural practice, and religious, cultural, or lifestyle considerations.

## Discussion

A study on compulsory community service for doctors in South Africa showed that 15% of community service doctors between 2000 and 2014 considered rural practice for the future.^8^ Our study shows a similar trend of interest, with 8% considering rural practice in 2023 and 20.9% considering it sometime in the future. This seems to suggest that any efforts in the last decade to encourage doctors to consider public rural practice have made minimal impact.^1,2,4,5,17^

Our study sample consisted of a predominance of Afrikaans and English-speaking community service doctors in the Western Cape. All, but four, participants were first language English or Afrikaans speakers with only three IsiXhosa-speaking participants. A study of medical officers in the Western Cape showed a similar language distribution, with 52.6% speaking Afrikaans and 44.2% speaking English.^25^ However, this distribution is not representative of the general population of the Western Cape. In 2016, 46.6% of the Western Cape’s population spoke Afrikaans as their home language, 19.6% spoke English and 31.1% spoke IsiXhosa.^26^ This may suggest a need for more isiXhosa-speaking doctors in the Western Cape public service, and this need is partly being addressed by the incorporation of isiXhosa (and Afrikaans) training in the undergraduate curricula of both the University of Cape Town and Stellenbosch University.^27^

Of the participants, 43% were married and 4% had life partners, which raises the question of whether consideration of a life partner is an important factor. In fact, the importance of ‘Factors influencing the quality of life of my partner’ yielded a mean score of 4.4 (very important to extremely important). Although only 10% of the participants had children, the ‘Factors influencing the quality of life of my children’, was ascribed some importance (3.81). Parenting and life partner-related factors were also found to be influential in a study on the retention of medical officers in the Western Cape district health services.^25^ In this study, being unable to live with their life partner had a statistically significant association (*p* = 0.04) with leaving the district health services.^25^ In a follow-up qualitative study, prioritising childcare was also a notable and important factor in determining retention in district health services.^28^ It is important to note that these studies did not measure whether the factors influenced decisions for or against rural employment, but all rather suggest that consideration of family is important to career decisions in general.

The large proportion of participants with undergraduate exposure to rural medicine (89.5%) suggests that training institutions are generally trying to expose their students to rural practice. This has been a widespread strategy globally which has shown some association with increased willingness to consider post-training placement in rural practice.^18^ However, for this study’s participants, this exposure does not seem to be a significant factor in their decision-making as it was ranked 30th out of 39 factors. On the other hand, a rural upbringing or rural internship or community service did show significant association with intention to work in rural practice. This supports a conclusion that while rural exposure is important, a rural upbringing is more significant than exposure during undergraduate training.^12,17,18^

The findings that rural upbringing promotes retention in rural practice support another strategy used in South Africa, namely, to train more doctors from rural and other underserved areas with the hope that they will return home to work.^29^ A specific example of this is offering medical training in Cuba to black students from disadvantaged communities.^29^ Only 10.5% of participants of this study grew up in rural areas. So increased efforts to recruit rural medical students are needed.

In this study, the participants subjectively ranked their rural upbringing as having low importance in their career decision. However, this demographic factor was significantly associated with a choice for future rural employment. A possible explanation for this ostensible contradiction may be that social ties such as family and community are tacitly or unconsciously significant in making this decision. This was demonstrated in studies on the migration of young adults from rural areas in Senegal and Europe.^30,31^ In the Senegalese study on migration, the decisions of young adults to stay in rural areas were influenced by family ties as seen by the positive association between being married and having children with decision not to migrate from their rural home.^30^ This may also explain the association between a rural internship and community service and future rural employment. Based on the Likert scale questions, the five least influential factors in this study include financial incentives (rural allowance), rural exposure during community service year, rural exposure during medical internship training, bursary obligations, and rural upbringing. The South African National Department of Health’s strategies to improve equitable access to healthcare human resources rely on these factors.^4,5^ For example, the Human Resources for Health Strategy for the Health Sector: 2012/2013 – 2016/2017 proposes the extension of rural exposure during training, creation of more posts and training of more healthcare workers.^5^ Other strategies include providing provincial financial support to trainees through bursaries and creating more training opportunities for people from underserved areas.^4,5,29^

The most influential factor in this study is crime and personal safety. However, this factor will have to be explored further as it is not clear from our study if crime and safety were a factor that was drawing people out of rural practice or making rural practice preferable. Furthermore, this question is not resolved in the current literature. A 2021–2022 survey found that more house break-ins occurred in rural areas, and most assaults occurred in ‘non-metro’ or non-urban areas.^32^ However, people in rural areas feel safer walking at night than their urban counterparts, although this feeling of safety in rural areas is on the decline.^29^

The next two most influential factors are job satisfaction and mental health. A systematic review of healthcare workers in sub-Saharan Africa showed that 81% of physicians at rural district hospitals in South Africa had experienced burnout.^19^ However, it is unclear whether the level of burnout in rural healthcare workers is higher than in urban healthcare workers. An American study found no difference in burnout between urban and rural family practitioners, while a Japanese study showed higher levels of burnout in rural hospital physicians.^33,34^ Factors associated with high levels of burnout included career dissatisfaction, heavy workload, work environment and inadequate staffing.^33,34^ The category in our study which had the highest average score was ‘Expected working conditions’. These are therefore important factors in the South African context and a good area to focus interventions for staff retention.

## Conclusions

This study found that despite strategies to promote a choice of career in underserved areas, the proportion of community service doctors considering working in public rural practice has not significantly increased (20%). The only demographic factors shown to have a significant association with an intention to work in public rural practice sometime in the future were a rural upbringing, having done internship and community service in rural areas. The most important factors influencing community service doctors’ decisions on where to work in future included concerns for personal safety and security, job satisfaction and their mental health. The practical implication of this is that a revision of strategy on the part of policymakers should be considered, and areas of focus should be promoting safe, satisfying work environments which are protective of staff mental and psychological well-being while continuing to preferentially enrol medical students with a rural upbringing and prioritise placing community service doctors in rural areas.

Based on the study findings, the following recommendations are proposed:

- Implement strategies that promote safe, satisfying working environments in rural hospitals to improve retention of doctors in public rural services.
- Institutions need to take the mental and psychological well-being of their employees seriously and should consider interventions to promote mental health and the prevention of burnout.
- Continued preferential selection of medical students from underserved rural areas by medical schools.
- Preferential placement of interns and community service doctors in rural institutions by the National Department of Health.
- Expand this study to a national level to provide meaningful, evidence-informed, national policy guidance. This larger study sample should yield more precise results and improve their generalisability.
- For any future similar study, review the questionnaire to consider the additional factors raised in the final open-ended question.
- A concurrent qualitative study may help to understand, in more nuanced detail, how to engage with the significant factors highlighted in our study.

### Limitations

The optimal response rate was not reached in this study. The small sample size and even smaller proportion of those considering careers in rural public practice in 2023 make identifying statistically significant associations difficult. Selection bias cannot be excluded, and non-response and/or volunteer bias affects this study’s generalisability in the Western Cape.

This study would not be generalisable to the rest of the country as it was performed in the Western Cape, which has a unique health system as well as population and urban-rural characteristics.

The high proportion of female participants may represent a higher tendency for female participants to respond to research surveys, thereby affecting the representativeness of the sample.^35,36,37^ However, this predominance may also be because of more female community service doctors being employed than male community service doctors. Unfortunately, the proportion of female community service doctors in the study population is not known.^29,30,31^

Lastly, the way the questionnaire was phrased did not specify if the factors were influencing people towards or away from rural or urban practice. It also did not separate or differentiate factors influencing retention at any facility from those influencing retention in rural facilities. Therefore, although the questionnaire identified important factors, further clarification on how they influence the decisions is still needed.

## References

[1] Weinhold I, Gurtner S. Understanding shortages of sufficient health care in rural areas. Health Policy (New York). 2014;118(2):201–214. 10.1016/j.healthpol.2014.07.018 25176511

[2] International Labour Office. Social Protection Department. Addressing the global health crisis: Universal health protection policies. Geneva: ILO, 2014; 50 p.

[3] Sirili N, Frumence G, Kiwara A, et al. Retention of medical doctors at the district level: A qualitative study of experiences from Tanzania. BMC Health Serv Res. 2018;18(1):260. 10.1186/s12913-018-3059-0 29631589 PMC5891935

[4] Health Systems Trust. South African health review 2020 [homepage on the Internet]. 2020 [cited 2022 Jun 21]. Available from: https://www.hst.org.za/publications/Pages/SAHR2020.aspx

[5] Department of Health Republic of South Africa. Human resources for health South Africa: HRH Strategy for the health sector: 2012/13 – 2016/17. Johannesburg, SA: National department of Health; 2011.

[6] Bradshaw D. Determinants of health and related indicators. In: Barron P, Roma-Reardon J, editors. South African health review 2008. Durban: Health Systems Trust, 2008; p. 23–36.

[7] Ayeni OA, Walaza S, Tempia S, et al. Mortality in children aged < 5 years with severe acute respiratory illness in a high HIV-prevalence urban and rural areas of South Africa, 2009–2013. PLoS One. 2021;16(8):e0255941. 10.1371/journal.pone.0255941 34383824 PMC8360538

[8] Reid SJ, Peacocke J, Kornik S, Wolvaardt G. Compulsory community service for doctors in South Africa: A 15-year review. S Afr Med J. 2018;108(9):741–747. 10.7196/SAMJ.2018.v108i9.13070 30182899

[9] Mohammadiaghdam N, Doshmangir L, Babaie J, Khabiri R, Ponnet K. Determining factors in the retention of physicians in rural and underdeveloped areas: A systematic review. BMC Fam Pract. 2020;21(1):216. 10.1186/s12875-020-01279-7 33097002 PMC7585284

[10] Esu EB, Chibuzor M, Aquaisua E, et al. Interventions for improving attraction and retention of health workers in rural and underserved areas: A systematic review of systematic reviews. J Public Health (Oxf). 2021;43(Suppl 1):i54–i66. 10.1093/pubmed/fdaa235 33856468

[11] Liu X, Dou L, Zhang H, Sun Y, Yuan B. Analysis of context factors in compulsory and incentive strategies for improving attraction and retention of health workers in rural and remote areas: A systematic review. Hum Resour Health. 2015;13:61. 10.1186/s12960-015-0059-6 26194003 PMC4508764

[12] MacQueen IT, Maggard-Gibbons M, Capra G, et al. Recruiting rural healthcare providers today: A systematic review of training program success and determinants of geographic choices. J Gen Intern Med. 2018;33(2):191–199. 10.1007/s11606-017-4210-z 29181791 PMC5789104

[13] Tawana B, Barkhuizen NE, Du Plessis Y. A comparative analysis of the antecedents and consequences of employee satisfaction for urban and rural healthcare workers in KwaZulu-Natal province, South Africa. SA J Hum Resour Manag. 2019;17(1):1–9. 10.4102/sajhrm.v17i0.1080

[14] George G, Atujuna M, Gow J. Migration of South African health workers: The extent to which financial considerations influence internal flows and external movements. BMC Health Serv Res. 2013;13(1):297. 10.1186/1472-6963-13-297 23919539 PMC3765273

[15] George A, Blaauw D, Thompson J, Green-Thompson L. Doctor retention and distribution in post-apartheid South Africa: Tracking medical graduates (2007–2011) from one university. Hum Resour Health. 2019;17(1):100. 10.1186/s12960-019-0439-4 31842879 PMC6916458

[16] World Health Organization 2020. Retention of the health workforce in rural and remote areas: A systematic review [homepage on the Internet]. [cited 2024 Feb 10]. Available from: https://www.who.int/news/item/08-12-2020-retention-of-the-health-workforce-in-rural-and-remote-areas-a-systematic-review

[17] Holloway P, Bain-Donohue S, Moore M. Why do doctors work in rural areas in high-income countries? A qualitative systematic review of recruitment and retention. Aust J Rural Health. 2020;28(6):543–554. 10.1111/ajr.12675 33197109

[18] Holst J. Increasing rural recruitment and retention through rural exposure during undergraduate training: An integrative review. Int J Environ Res Public Health. 2020;17:1–19. 10.3390/ijerph17176423 PMC7503328 32899356

[19] Dubale BW, Friedman LE, Chemali Z, et al. Systematic review of burnout among healthcare providers in sub-Saharan Africa. BMC Public Health. 2019;19(1):1247. 10.1186/s12889-019-7566-7 31510975 PMC6737653

[20] Kotzee TJ, Couper ID. What interventions do South African qualified doctors think will retain them in rural hospitals of the Limpopo province of South Africa? Rural Remote Health. 2006;6(3):581. 10.22605/RRH581 16965219

[21] Hain S, Tomita A, Milligan P, Chiliza B. Retain rural doctors: Burnout, depression and anxiety in medical doctors working in rural KwaZulu-Natal Province, South Africa. S Afr Med J. 2021;111(12):1197–1204. 10.7196/SAMJ.2021.v111i12.15841 34949307

[22] Baytopp T, Motsohi T, Lomas V. Factors influencing Western Cape community service doctors’ choice of whether to seek employment in public, rural practice [homepage on the Internet]. 2024 [cited 2024 Nov 20]. Available from: https://scholar.sun.ac.za/server/api/core/bitstreams/4eae167c-0b88-410f-a6e8-5413659279a9/content

[23] Statistics South Africa. Census 2001: Investigation into appropriate definitions of urban and rural areas for South Africa: Discussion document. Pretoria: Statistics South Africa, 2003; 187 p.

[24] Western Cape Government Health. Western Cape health annual report 2020/2021 [homepage on the Internet]. 2021 [cited 2022 May 23]. Available from: https://www.westerncape.gov.za/assets/annual_report_2020_2021_0.pdf

[25] Mash R, Williams B, Stapar D, et al. Retention of medical officers in district health services, South Africa: A descriptive survey. BJGP Open. 2022;6(4):BJGPO.2022.0047. 10.3399/BJGPO.2022.0047 36167403 PMC9904795

[26] Provincial profile: Western Cape Community Survey 2016 [homepage on the Internet]. 2018 [cited 2022 May 23]. Available from: cs2016.statssa.gov.za/?portfolio_page=community-survey-2016-provincial-profile-western-cape-2016

[27] Claassen J, Jama Z, Manga N, Lewis M, Hellenberg D. Building freeways: Piloting communication skills in additional languages to health service personnel in Cape Town, South Africa. BMC Health Serv Res. 2017;17(1):390. 10.1186/s12913-017-2313-1 28592265 PMC5463348

[28] Mash RJ, Viljoen W, Swartz S, et al. Retention of medical officers in the district health services of the Western Cape, South Africa: An exploratory descriptive qualitative study. S Afr Fam Pract. 2022;64(1):13. 10.4102/safp.v64i1.5467 PMC9210154 35695448

[29] Sui X, Reddy P, Nyembezi A, et al. Cuban medical training for South African students: A mixed methods study. BMC Med Educ. 2019;19(1):216. 10.1186/s12909-019-1661-4 31208423 PMC6580452

[30] Schewel K. Working Papers Understanding the aspiration to stay a case study of young adults in Senegal. The IMI Working Papers Series [homepage on the Internet]. 2015 [cited 2022 May 23]. Available from: www.researchgate.net/publication/324067928_Understanding_the_Aspiration_to_Stay_A_Case_Study_of_Young_Adults_in_Senegal

[31] Stockdale A, Theunissen N, Haartsen T. Staying in a state of flux: A life course perspective on the diverse staying processes of rural young adults. Popul Space Place. 2018;24(8):e2139. 10.1002/psp.2139

[32] Experience of crime in SA increased over the 2021/22 period [homepage on the Internet]. Statistics South Africa. [cited 2023 Jul 28]. Available from: https://www.statssa.gov.za/?p=15700

[33] Saijo Y, Chiba S, Yoshioka E, et al. Job stress and burnout among urban and rural hospital physicians in Japan. Aust J Rural Health. 2013;21(4):225–231. 10.1111/ajr.12040 24033524

[34] Ward ZD, Morgan ZJ, Peterson LE. Family physician burnout does not differ with rurality. J Rural Health. 2021;37(4):755–761. 10.1111/jrh.12515 32929816

[35] Bälter KA, Bälter O, Fondell E, Lagerros YT. Web-based and mailed questionnaires: A comparison of response rates and compliance. Epidemiology. 2005;16(4):577–579. 10.1097/01.ede.0000164553.16591.4b 15951679

[36] Tiwari R, Wildschut-February A, Nkonki L, English R, Karangwa I, Chikte U. Reflecting on the current scenario and forecasting the future demand for medical doctors in South Africa up to 2030: Towards equal representation of women. Hum Resour Health. 2021;19(1):27. 10.1186/s12960-021-00567-2 33653366 PMC7923812

[37] Breier M, Wildschut-February A, Wildschut A. Changing gender profile of medical schools in South Africa. S Afr Med J [serial online]. 2016 [cited 2024 Feb 13]. Available from: https://www.researchgate.net/publication/23252549 18785399

